# Perceptions of Wearable Health Tools Post the COVID-19 Emergency in Low-Income Latin Communities: Qualitative Study

**DOI:** 10.2196/50826

**Published:** 2024-05-08

**Authors:** Stefany Cruz, Claire Lu, Mara Ulloa, Alexander Redding, Josiah Hester, Maia Jacobs

**Affiliations:** 1 Department of Electrical and Computer Engineering McCormick School of Engineering Northwestern University Evanston, IL United States; 2 Department of Computer Science McCormick School of Engineering Northwestern University Evanston, IL United States; 3 Department of Computer Science and Engineering Irwin & Joan Jacobs School of Engineering University of California, San Diego La Jolla, CA United States; 4 Interactive Computing and Computer Science College of Computing Georgia Institute of Technology Atlanta, GA United States; 5 Department of Preventative Medicine, Feinberg School of Medicine Northwestern University Evanston, IL United States

**Keywords:** mHealth, mobile health, wearable, wearables, Health wearables, COVID-19, digital divide, low-socioeconomic status, socioeconomic, adoption, underserved, poverty, low income, low resource, marginalized, equity, attitude, attitudes, opinion, opinions, perception, perceptions, perspective, perspectives, acceptance, Spanish, Hispanic, Latinx, Hispanics, interview, interviews

## Abstract

**Background:**

Mobile health (mHealth) wearable devices are increasingly being adopted by individuals to help manage and monitor physiological signals. However, the current state of wearables does not consider the needs of racially minoritized low–socioeconomic status (SES) communities regarding usability, accessibility, and price. This is a critical issue that necessitates immediate attention and resolution.

**Objective:**

This study’s aims were 3-fold, to (1) understand how members of minoritized low-SES communities perceive current mHealth wearable devices, (2) identify the barriers and facilitators toward adoption, and (3) articulate design requirements for future wearable devices to enable equitable access for these communities.

**Methods:**

We performed semistructured interviews with low-SES Hispanic or Latine adults (N=19) from 2 metropolitan cities in the Midwest and West Coast of the United States. Participants were asked questions about how they perceive wearables, what are the current benefits and barriers toward use, and what features they would like to see in future wearable devices. Common themes were identified and analyzed through an exploratory qualitative approach.

**Results:**

Through qualitative analysis, we identified 4 main themes. Participants’ perceptions of wearable devices were strongly influenced by their COVID-19 experiences. Hence, the first theme was related to the impact of COVID-19 on the community, and how this resulted in a significant increase in interest in wearables. The second theme highlights the challenges faced in obtaining adequate health resources and how this further motivated participants’ interest in health wearables. The third theme focuses on a general distrust in health care infrastructure and systems and how these challenges are motivating a need for wearables. Lastly, participants emphasized the pressing need for community-driven design of wearable technologies.

**Conclusions:**

The findings from this study reveal that participants from underserved communities are showing emerging interest in using health wearables due to the COVID-19 pandemic and health care access issues. Yet, the needs of these individuals have been excluded from the design and development of current devices.

## Introduction

### Background

As mobile computing has advanced, wearable technologies have become more ubiquitous; however, in their current state, wearables threaten to worsen digital and health inequities and perpetuate structural harm in the health care sector and society as a whole [1,2]. Health disparities persist that limit the positive health outcomes of those from low–socioeconomic status (SES) backgrounds. The inequitable allocation of resources prevents many from low-SES communities from accessing quality health care, and systemic racism continues to pervade the health care system on both an individual provider and more macro level [3-6]. Further, the social determinants of health, which include factors such as economic status and race, are now understood to be the primary drivers of health outcomes, meaning that health disparities can manifest from societal structures even before an individual interacts with the health care system [7,8]. As a result, current systems fail to adequately provide for the health of Latine and low-SES communities, contributing to the preventable higher rates of illness and death among these groups.

Wearable devices are electronic smart devices delivered in a range of form factors including accessories and clothing that can sense biological and environmental factors and perform influential predictive computations. Most recent wearables, such as the Apple Watch (Apple Inc), have opened new avenues for capturing important physiological data such as measuring heart rate, tracking sleeping, and capturing electrocardiogram data. While wearables have demonstrated their potential to improve the health of low-SES communities by helping them increase physical activity through fitness trackers [9], research shows that wearable devices have primarily been designed with the participation of more affluent communities, and low-SES communities have historically been and continue to be excluded from their design [2-6]. The exclusion of low-income communities in technological design can have unintended and harmful consequences [10]. For example, the photoplethysmography sensor, commonly used for measuring heart rate, detecting arrhythmia, and tracking sleep, has been shown to be less accurate or fail to function properly for individuals with darker skin tones [11,12]. Given that poverty rates are over twice as high for Black and Hispanic or Latine or Latinx groups (hereby referred to as Latine) in comparison to White individuals [13,14] inaccurate readings from a photoplethysmography sensor may disproportionately impact low-SES communities. This is particularly troubling, given that our research participants all identified as members of low-income Latine communities. There is a critical need for wearables to overcome existing access and accuracy issues for these marginalized communities and to be developed directly with community members to ensure goal and value alignment.

### Objectives

The development and design of current health wearables have predominantly been driven by the experiences of affluent communities. Consequently, there is a need to collaborate with low-SES community members to better understand the potential of wearables for addressing their health needs, goals, and experiences. Through an exploratory qualitative research study, we aimed to understand (1) how members of low-SES communities perceive mobile health (mHealth) wearable devices and (2) identify the barriers and facilitators toward adoption. The results point to requirements and recommendations for designing and developing better mHealth wearable devices more equitably, enabling low-SES community members to understand and self-manage their health and well-being.

## Methods

### Study Design

This exploratory qualitative study aimed to identify low-income community members’ perspectives and needs toward wearable devices. This study was part of a larger project investigating low-SES community members’ perspectives of wearable devices and the barriers and facilitators toward adoption [15]. We conducted 19 semistructured interviews with members of low-SES Latine communities from 2 metropolitan cities in the United States. Interviews were conducted separately in 2 different rounds. Our initial interviews, consisting of 8 participants, aimed at understanding low-SES community members’ perspectives on wearable technology more broadly to obtain an understanding of community members’ needs and expectations. Though questions on the impacts of COVID-19 and its relation to wearable devices were not asked, the topic was brought up by participants so frequently that we felt the topic warranted more in-depth discussion. Therefore, we ran a second round of interviews with 11 additional participants, in which questions were focused on participants’ perspectives on how mHealth wearables can support them in their everyday lives. This research study was conducted from December 2021 to March 2022. Due to the COVID-19 Omicron variant, all interviews were conducted in English over a Zoom (Zoom Video Communications, Inc) video call. The results are reported following the Standards for Reporting Qualitative Research by O’Brien (see Multimedia Appendix 1) [16].

### Recruitment

We were interested in working with low-SES community members in the United States. Flyers containing information about this study and a link to a Qualtrics (Qualtrics) screening survey were posted on the research team’s social media sites, such as Facebook and Instagram (Meta Platforms). Interested participants completed a screening survey that asked for basic demographic information (eg, race, education level, household income, and the number of persons in the household). The inclusion criteria were determined if participants identified as (1) aged older than 18 years, (2) BIPOC (Black, Indigenous, and people of color), and (3) of low-income. Criteria 3 was met if an individual’s income level fell at or below the low-income threshold according to their county’s Department of Housing and Community Development (in the United States, the Department of Housing and Community development uses State Income Limits provided by the US Department of Housing and Urban Development [17]). Individuals were excluded if they did not meet all the above criteria. After an eligible participant signed a digital consent form (see Multimedia Appendix 2), an online semistructured interview session was scheduled. We note that all participants came from 2 metropolitan cities in the United States but being from a particular city or location was not part of the eligibility criterion.

### Study Procedure

#### Overview

Interview sessions were conducted by the lead author. All interviews were 45 to 60 minutes in length and were conducted over a Zoom video call. In these interview sessions, we sought to learn about participants’ opinions regarding wearable technology for health.

A preliminary round of interviews centered on exploring participants’ access to Wi-Fi connectivity, technology, and any resource constraints they may encounter was conducted. Additionally, we delved into their perspectives on wearable technology, their community’s perception of wearables, their likes and dislikes about current wearable devices, and the features they would like to see in future wearable devices; interview questions from the preliminary study can be found in Multimedia Appendix 3.

A second round of interviews was initiated to focus on participants’ views of mHealth wearable devices, their opinions on the general health and well-being of people in their neighborhood and community, the impact of COVID-19 in their community, and the types of health information considered by participants to be useful for them. The participants were asked if they had an idea what a wearable device was, and if they were unfamiliar, the interviewer described a brief definition and example. Interview questions from the second part of this study can be found in Multimedia Appendix 4. Interviews were halted once saturation was reached. All interview sessions were audio recorded and participants were compensated with a US $40 gift card at the end of the interview.

#### Data Analysis

Audio recordings of the interviews were transcribed, resulting in a total of 21 hours of interviews. During the preliminary investigation, the authors performed open coding on the transcripts and analyzed the data using a grounded theory approach, following the methods defined by Charmaz and Belgrave [18]. Further, 2 members of the research team performed open coding on the transcripts and identified initial themes. The research team then reviewed the transcripts and collaboratively discussed associated codes to look for consistencies and differences in the data. Through a collaborative process involving group discussions, an iterative refinement of themes was conducted. Among the emerging themes, one stood out as particularly significant: the relationship between health and wearables in the context of COVID-19. This theme was subsequently used to develop the interview guide for the second round of interviews.

In the secondary investigation, the lead author and fourth author once again performed open coding on the transcripts and analyzed the data using a grounded theory approach. Author 3 was added to the research team to help identify and narrow themes. The research team once again reviewed the transcripts and collaboratively discussed associated codes to look for consistencies and differences in the data. Codes were merged into subthemes and then grouped into 4 prominent themes. Consensus was reached by involving the sixth author to determine the final themes.

#### Research Reflexivity

The research team consisting of the first, third, and fourth authors are part of Latine low-SES communities. The first author, a doctoral student, recruited participants, designed this study, conducted the semistructured interviews, and analyzed the transcripts. The third and fourth authors are doctoral students and helped collect and analyze the data. All other authors contributed to drafting and revising this paper.

#### Ethical Considerations

This project’s study protocol was reviewed and approved by Northwestern University’s institutional review board (STU00216152).

## Results

### Participants

We recruited 19 adults from low-SES communities in 2 metropolitan cities in the United States. In total, 8 participated in the first round of interviews and 11 in the second round. All participants identified as Hispanic or Latinx and had low income. Additionally, 2 participants identified as members of the LGBTQ+ (lesbian, gay, bisexual, transgender, and queer) community. Furthermore, 94.7% (18/19) of the participants were from Los Angeles and 5% (1/19) of the participants were from Chicago. Participants’ ages ranged from 18 to 54 (mean 29.7, SD 10.81) years. Table 1 summarizes participant demographics.

**Table 1:** Sample characteristics (N=19).

Characteristics and variable or category		Participant, n (%)
**Sex**		
	Female	14 (78.9)
	Male	5 (21.1)
**Age (years)**		
	18-29	12 (63.2)
	30-44	6 (31.5)
	45-54	1 (5.26)
	≥55	0 (0)
**Education**		
	<High school	0 (0)
	High school	3 (10.5)
	Two years or some college	8 (42.1)
	Bachelors	8 (42.1)
	Graduate school	0 (0)
**Income range (US $)**		
	<26,000	7 (36.8)
	26,000-50,000	9 (47.4)
	50,000-75,000	3 (15.8)
	75,000-100,000	0 (0)
	<100,000	0 (0)
**Owns wearable**		
	Apple Watch	5 (26.3)
	Samsung Galaxy Watch (SAMSUNG)	1 (5.26)
	Fitbit (previously; Google)	2 (10.5)

### Themes

#### Overview

Four major themes emerged from our data analysis: (1) how COVID-19 changed community members’ interest in wearables for health; (2) barriers to health care resources, seeking alternatives through wearables, (3) distrust in the medical system, motivating the needs for wearables as a potential solution; and (4) community-based technical requirements. All participants said they were aware of wearable technology. We discuss our results in more depth in the following sections.

#### Theme 1: How COVID-19 Increased Interest in Personal Health Monitoring Through Wearables for Health

The impact of COVID-19 on underrepresented communities was significant and had a clear and direct effect of exacerbating existing health disparities [19]. Even though the COVID-19 public health emergency has ended, the residual effects of the pandemic have left individuals with long-term symptoms.

Participants (n=14) shared that they themselves had been infected with COVID-19 and had long-lasting health problems as a result. Participants (n=7) mentioned that they were still experiencing breathing problems despite having no health issues before being infected. Further, 1 participant elaborated as follows:

I got COVID the first time, right when the pandemic started... Afterwards it was hard for me to breathe when walking... The second time around that I got it, which was recently, it hit me a lot harder. Before I got COVID, I was healthy like there was nothing wrong with me. So, it's definitely taking its toll. I was used to walking 5 miles a day. I was walking 20 something miles a week so you know it wasn't normal for me to have breathing problems.

As a result of experiencing ongoing COVID-19 symptoms, participants shared that for the first time, they are considering how health data, such as monitoring their oxygen levels, could be helpful. This led to conversations about how participants wished they had wearable tools that automatically measured their oxygen levels and allowed them to monitor the data themselves. Participants expressed the following:

I guess the one thing that scares me that I never even thought of until I got COVID were like my oxygen levels. Like, am I at normal levels? Is that an issue that I need to kind of think about, you know?

Another participant commented:

One thing I noticed, especially with COVID right now which is, I think a very big topic. The timing of getting all your vitals measured can actually save somebody's life. So, I think that's a very important thing. Like oxygen levels to be measured.

Through these interviews, we uncovered overwhelming interest in the types of data that can be provided through health wearables, primarily as a result of the long-term consequences of COVID-19 and resulting interest in engaging in personal health monitoring.

#### Theme 2: Barriers to Health Care Resources, Seeking Alternative Health Monitoring Through Wearables

Participants frequently discussed a severe lack of health resources and infrastructure in their communities, made worse by COVID-19, and expressed interest in potential alternatives to manage their health care through wearables to compensate for the lack of local health resources. Specifically, participants shared that the hospitals in their neighborhoods were shut down, forcing community members to seek health care in small clinics. A participant explained:

Most Hispanics don't have health care. I do not have that great health care myself and I have two jobs ... We lost, we had a hospital down the street, and they went bankrupt. Right now, all we really have is small clinics. So, I'm pretty sure that's all the help that anybody around here can get and it’s really busy.

Many participants (n=11) mentioned that these small clinics were completely inaccessible due to overcrowding.

It's overly populated. Even if you make an appointment, you're there all day. Whatever time you go, whatever day you go, it's always crowded, because it's one of the very few [clinics] that accepts Medi-Cal. So low-income communities, they don't have the resources, it's always crowded.

Lack of health care coverage was another stressor all participants cited in conversation. Participants mentioned they could not be seen or treated and were left to fend for themselves. Individuals who experienced more critical symptoms were not able to receive treatment and passed away.

Health coverage was a very big issue, because not everybody was able to afford to, let's say, be able to get seen or get treated. A lot of stuff was very limited, especially to the community. If you got sick, well, your best guess to do was rub VapoRub on yourself cause that's what we only have. … A lot of these people didn't get treated and passed [away].

Many participants felt the impact of COVID-19 could have been mitigated if the proper health care infrastructures were implemented. However, the idea of having wearables that can potentially measure vital signs and other health parameters that do not require doctor visits came up often in conversations as a practical alternative. Participants shared how they felt wearables could be useful for individuals who live in underserved areas and who do not have access to the proper health care infrastructure.

If [the wearable] was easy for people to use then for sure. Just knowing when people who are in areas that need more resources or who need more hospitals. Like knowing what's going on with people without having them come into the hospital to find out, because how often do people really go to the clinic and get their vitals checked, so just maybe having an idea of what is going on with people beforehand, that would be really cool.

Thus, we found that undersourced and poor health care infrastructure has led participants to express interest in using wearable technologies for health self-management.

#### Theme 3: Distrust in Health Care Infrastructure and Systems Is Motivating a Need for Wearables

We found that community members’ distrust of their health systems increased their interest in health wearables. Participants described experiences that led to their mistrust and fear of the health care system. For example, being turned away from immediate hospital care resulted in a participant’s family member’s medical conditions deteriorating leading to their death.

My uncle, that's another issue, they didn't want to give him a covid test for some reason and he just stayed at home for like 2-3 weeks feeling sick and only [when]he had very large symptoms he [got seen at] the hospital, and he died there two days later.

The participants expressed that inadequate infrastructure and being denied medical assistance have made digital health care tools, like wearables, a valuable option for health testing and monitoring. The participants desired tools that could enable them to receive timely care and prevent late hospital visits. A participant highlighted that community members’ lack of trust in doctors, coupled with high medical expenses, made seeking medical help unfeasible. Nonetheless, they suggested that this distrust in the health system could motivate people to take charge of their health and use wearable devices to conduct essential vital tests, rather than relying solely on medical professionals.

I think if you're worried about a wearable device running tests on people, cause let's be honest. Hispanic people don't go to the doctor because they don't believe in the doctor. They think the doctors are gonna kill them and then they're poor, so they can't pay for the doctor. So, like if [a wearable] could do basic [vital] tests that would be great.

Participants suggested that wearables could offer patients a new avenue to engage in health care, especially in cases where conventional medical services are not accessible or when patients are hesitant to rely on data collected by their physicians. By using wearables instead, users can set and track their own health goals through data, alleviating dependence on the health care system to gain ownership over personal preventative health strategies. Consequently, participants also expressed a desire to have ownership of their health data and to better understand the significance of this information for their personal health.

I think the biggest benefit to wearables will probably be the health aspect, like if you want to be constantly measuring your heart rate or measuring how many steps you walked in a day, I think that's where those devices will be very useful.

Further, 17 other participants echoed this comment and expressed enthusiasm for having the ability to track health data that is important to them. Additionally, 18 out of the 19 participants expressed a strong interest in adopting wearables to support their health goals. Only 1 participant felt that they did not see the benefits of current wearables, specifically because they felt they could cheat on how their physical activity is being tracked by moving their hand around instead of moving to obtain their steps. Table 2 provides an overview of the most sought-after health parameters for monitoring. Notably, we observed that individuals from low-SES communities demonstrated a heightened interest in wearables and identifying the most critical health information to track.

**Table 2:** Signals participants (N=19) want to capture through wearable sensors.

Signal category	People, n
Heart rate sensors	18
Physical activity	17
Oxygen levels	13
COVID-19 symptoms	11
Blood pressure	11
Diabetes	10
Vitals	10
Breathing	7
Temperature	5
Stress	4
Glucose	4
Mental health	3
Cholesterol	3
Blood sugar	3
Allergies	1

#### Theme 4: Community-Based Technical Requirements

Participants shared several contextual considerations on how wearables can be designed to meet the needs of marginalized groups. The most common technical requirements emphasized throughout the interviews were durability, autonomy over data, and affordability.

#### Durability for Employment Reasons

As mentioned earlier, participants live in communities where most of the population identify as Hispanic or Latinx and they tend to work in more physically demanding environments. Hence, a common preference participants discussed if a wearable device were to be designed to meet the constraints of low-income communities was durability. A participant elaborated on the importance of durability:

I do think that it has to be very durable because the purpose is [for] low income communities. They don't have money to replace it. We just don't have comfy jobs. A lot of us work more physically demanding jobs. Some of us are plumbers, some are construction workers, some of us are gardeners. Some of us run a business and like that business involves pots and pans like we're restaurant workers. If [the device] breaks, they're just gonna say oops and throw it away. Or they're gonna like cry about it and be really upset. And they're like how do I pay for this again, you know? If it is more durable that’s one of the biggest keys to wearing it.

Participants demonstrated a significant inclination toward health wearables, specifically those that monitor vital signals such as oxygen levels. However, since such wearables must be worn continuously throughout the day, their durability becomes a critical factor. Therefore, it is imperative to ensure that these wearables are built to last and withstand the rigors of daily wear.

#### Autonomy: Would Rather Have Control Over Data

Privacy and having control over their own data was another critical component participants discussed. Participants did not feel comfortable having their data shared with big tech companies or a single health system due to the mistrust of both entities:

I think that's actually one of the biggest things that stops me from getting these, ‘smart stuff.’ It can measure your heart rate, has your location and all that. That sounds cool but for me my concern is how do I know if they're not sending it to a server? If it's being sent to server and I wouldn't feel comfortable with [tech companies] having access to all that data and just knowing where I'm going, what my vitals are that’s just for me a bit Orwellian. I think the big thing for me is feeling comfortable and having control [over data].

Participants shared conflicting viewpoints on this topic. For instance, 1 participant said:

People will be skeptical, you know they'll say, ‘oh, now they're gonna read my mind or something’. Especially in low-income communities, because unfortunately we don't have the same education. A lot of people rush to conclusions and say ‘ oh, that's not real’ or ‘they're just trying to track me’. So I am thinking from that point of view, because I do have relatives that think that way. But at the same time, you always have the batch of people who are like, well, you know, let's try this [technology].

Overall, these findings highlight diverse opinions regarding privacy and information sharing with big tech companies.

#### Affordability: Can Health Insurance Cover the Cost of Wearable Devices?

All participants mentioned that affordability was a barrier to adopting wearable devices, in particular wearables that are designed for health purposes.

I feel it's really limited because you either get a cheaper wearable technology and it's just not that advanced or not as reliable or you get expensive wearable technology that is honestly out of your price range. We would have to save up for it. And if you don't have access to like insurance then it sucks. My mom doesn't have access to one of those skin sugar sensors they have come out with. because insurance just doesn't cover it… wearable technology it definitely works, it's just expensive.

Community members often face a challenging trade-off between functionality and affordability. Further efforts are necessary to cultivate innovative strategies that can ensure the reliability and affordability of such devices. Participants highlighted the potential health impacts of wearable devices on their lives, but they also expressed concerns about the way wearables are currently designed. Table 3 summarizes our study’s findings and implications.

**Table 3:** Summary of key findings and implications.

Theme	Summary of participant needs	Recommendations in technical solutions moving forward
Theme 1: how COVID-19 increased interest in personal health monitoring through wearables for health	Increased interest in tools to monitor health signals through wearables to monitor long-term COVID-19 symptoms.	Hardware: develop embedded electronic sensors in mobile health and clinical tools that do not perpetuate racial disparities. Software: algorithms deployed for signal processing and machine learning must not perpetuate bias.
Theme 2: interest in self-checking vital signs in response to health care access barriers	In light of systemic challenges, participants wanted alternatives to self-manage their health care through wearables when limited resources are available.	Develop low-cost, low-power sensors that are still capable of measuring physiological signals robustly. Repurpose and upcycle existing hardware components. Open-source hardware and software for transparency and reliability. Increase service life for affordability and repairability.
Theme 3: distrust in health care infrastructure and systems is motivating a need for wearables	Participants expressed a desire to use health wearables to set and monitor personal health goals, with a focus on preventive health measures, such as increased exercise.	Develop wearables that robustly measure important physiological signals (as shown in Table 2) and allow individuals to self-manage these health parameters.
Theme 4: community-based technical requirements	Participants require durability due to physically demanding blue-collar jobs. Participants held varying opinions on privacy and the sharing of their health data with large tech corporations. Cost was the most commonly discussed barrier to adoption.	Wearable devices must be long-lived, durable, and adaptable for individuals who work in occupations that demand physical labor. Wearables should allow users to have autonomy over how their data are disseminated. Enhancing the affordability of wearables is crucial to ensure wider accessibility.

## Discussion

### Principal Findings

#### Overview

This study examined how members of minoritized low-income communities perceive mHealth wearable devices, what their needs and preferences are for using wearable devices, and understood the important contextual considerations for designing wearables in these communities.

The participants of this study expressed a newfound interest in health wearables for addressing their most critical health needs. Participants expressed a need for wearable devices that enable them to track vital signs and longitudinal symptoms (particularly those related to COVID-19) for setting and achieving preventive health goals, as illustrated in Table 2. Additionally, the participants emphasized that the wearable devices must be durable, reasonably priced, and within their budget or covered by their insurance, particularly state-provided insurance. It is evident from our study that the significant consequences of the COVID-19 pandemic on low-SES communities have led to an increased interest in health engagement and, subsequently, health wearables. However, current wearable devices fail to account for these individuals’ lived experiences. Below, we elaborate on this, discussing how mHealth wearable devices can be better designed to improve the health care experience of low-income community members by integrating with medical systems, increasing autonomy, and empowering them to make informed decisions about their care.

#### Overcoming Technology Limitations

Though participants expressed strong interest in using wearable devices to improve and promote their understanding of their health, existing wearables are not currently built to meet their needs.

According to Table 2, monitoring heart rate, physical activity, and oxygen levels were the top 3 areas of interest for participants. However, current commercial wearables’ optical sensors and the signal processing methods used to measure physiological signals are not reliable on darker skin tones [11]. This is a troubling concern because our study’s participants identify as Latine or Latinx, which are a minoritized group of color, are members of low-SES communities, and they stand to be disproportionately affected by inaccurate photoplethysmography sensors.

Photoplethysmography sensors are widely used in pulse oximeters to measure blood oxygen saturation levels. However, earlier research has demonstrated that the accuracy of pulse oximeters decreases when used on individuals with darker skin [19-22]. Sjoding et al [22] highlighted the dangers of relying on pulse oximeters, particularly during the COVID-19 pandemic. Patients who identified as Black were three times more likely to be incorrectly classified as having normal oxygen levels in the blood than their White counterparts. Such misclassification can result in a failure to detect hypoxemia (low oxygen levels in the blood), which can lead to severe health consequences. To address this issue, the Food and Drug Administration has cautioned against using pulse oximeters on individuals with dark skin tones, thick skin, poor circulation, and other factors that can impact the precision of the results [23].

Given the significant limitations of this technology, we see a clear need to (1) develop new embedded electronic sensors in mHealth and clinical tools that do not perpetuate racial disparities and (2) in software, we must ensure that the algorithms deployed for signal processing and machine learning do not perpetuate bias.

Regarding the development of new sensors, a couple of avenues of research hold promise. In light of the complications found in photoplethysmography sensors, researchers are now looking at multiwavelength photoplethysmography signals to target a deeper range of measuring blood pressure and other cardiovascular parameters from the skin and show potential for being implemented in future wearables [24]. Single-channel bioimpedance [25] is another method being investigated by researchers and shows promise for potentially being implemented in future wearable devices. The key to making these hardware modifications usable in future wearable devices and minimizing racial bias is to diversify subject testing and include individuals with a broad spectrum of skin tones.

The signal processing, machine learning, and high-level algorithmic approach to prediction from these sensors must be considered at the software level so as not to perpetuate racial bias. Software mitigations for poor sensor resolution and precision are commonplace in critical systems—similar approaches are needed here. Wearable devices have the potential to perpetuate racial bias unless addressed. Many machine learning approaches to things like recidivism prediction for parolees [26], mortgage loans [27], and facial recognition [28] have already proven that without care and attention in their design and training, these software systems perpetuate racial discrimination among racially minoritized individuals. Software and firmware developers of wearables must ensure that the algorithms they deploy to capture data from biomedical sensors do not perpetuate these racial harms. Our study extends this work by establishing wearable devices as an important application for these sensor and software developments. By leveraging more equitable research on both the hardware and software level, we can address the health concerns exacerbated by the COVID-19 pandemic.

#### Durability and Adaptability for Diverse User Groups

Designing wearables acutely attuned to resource-constrained populations requires ensuring that the tools are both durable and scalable. As mentioned by participants, if wearables are being designed with low-income community members in mind, they must be made more durable as most individuals from these communities work in “more physically demanding jobs.”

Taking into consideration their income and employment concerns, we see a need to ensure that wearables built to serve the general public also scale to this already underserved subset of the broader population. This is particularly important since literature has shown that technology that serves broader populations may not scale to subgroups [29,30].

Our findings point to the need to rethink the development of wearable technology. Strategies for improving the durability and affordability of wearable devices are to develop and make use of low-cost low-power sensors that are still capable of robustly sensing physiological signals, reuse and upcycle existing materials such as hardware components, increase service life by allowing wearable devices to be repairable inexpensively, open-sourcing hardware and software would allow wearables to be more affordable because people will not have to pay for licensing fees, and exploring different form-factors of wearable design beyond smartwatches. We argue that devices that can withstand the pressures of the everyday difficulties faced by our study population would better serve them and be a tool by which teams building wearables encode the values and preferences of underserved populations, thus promoting health equity.

#### Autonomy Over Data

In contrast to previous research where test subjects claimed they would like to share their data with health care providers [31], participants in our study were skeptical about sharing too much information with their health care providers due to a lack of trust in the health care system and fear of being monitored by big technology companies. Prior research discusses that the distrust of technology and the medical system among racialized low-SES communities is not new and increases barriers to adopting technology [32-34]. However, in our study, most participants believed that they should have more control over how their data is used and shared. Thus, we see interesting and important tension in participants’ desire to use health technologies but concern about using them in partnership with health care systems due to a history of distrust. This points to important future research on how such tools might be able to foster patient or provider collaboration and communication within these communities where such distrust is prominent.

Serious privacy concerns regarding the data collected by wearable devices have also been reported and highlight the lack of protection of consumers’ data [35]. Health insurance and tech companies such as Aetna and Apple have partnered to offer Apple watches at a discounted price; customers have to meet their fitness goals in 24 months or pay the full price if goals are not met [36]. The implications of these partnerships between insurance companies and tech companies raise privacy concerns as the health data collected by wearables allow insurance companies to determine which customers seem profitable and can raise the premium rates or deny insurance for others who are not meeting their fitness goals [37]. Apart from the privacy concerns, the potential consequences of insurance companies using data collected by wearable devices to raise insurance rates or deny health insurance to customers can lead to further health disparities for individuals from low-SES communities who may not have the funds to purchase the device even at a discounted price or have the ability to meet their fitness requirements due to other family and work responsibilities. Members of low-SES communities are already being denied access to health care, wearables should not be used as another tool to perpetuate health disparities among these populations.

Studies on contact tracing [38,39], COVID-19 mobile apps [40], and health informatics adoption among low-income individuals [32,41] have shown that data privacy is important to general users. This is especially true for low-income populations due to their mistrust of the health care system. Previous research in human-computer interaction has demonstrated the feasibility of creating privacy-preserving tools for mobile apps [42,43], software tools [44-46], and Internet of Things devices [47-49] that help users retain control and autonomy over their data.

Despite the significant progress in disseminating privacy tools to the general public, these tools have yet to be extended to the wearable space. Therefore, further research is necessary to develop privacy-focused tools for wearables, such as mobile apps or wearable-based interfaces, that enable individuals to manage and comprehend their data flow. Additionally, these privacy tools must be accessible and user-friendly to promote fair use among underserved communities.

Potential solutions for addressing privacy concerns call for stronger privacy regulations and encouraging tech companies to be more transparent about their data collection and usage. Additionally, individuals must have the autonomy to selectively share data that they feel comfortable disclosing to health care providers and tech companies. This can be facilitated through user-centric mobile apps or wearable interface privacy tools that empower people to understand and manage the flow of their data.

Beyond addressing participants’ privacy concerns and data autonomy needs, we see an interesting and important tension between participants’ desire to use health technologies and concern about using them in partnership with health care systems due to a history of distrust. While health wearables provide tools that can be empowering and engaging for patients, they are not meant to circumvent health care systems or medical care. We see a critical need for future research on how such tools might be able to foster patient or provider collaboration and communication within these communities where such distrust is prominent.

#### Affordability

The 18 participants who expressed interest in adopting wearables in their daily lives all mentioned that affordability was the strongest barrier toward adoption. These findings are in line with previous works [1,15,31]. Of participants who already owned a wearable device, 3 mentioned that they were gifted with the device and would not have been able to afford one otherwise. The remaining participants who already owned a wearable reported that they either “*bought it during a Black Friday sale*” or saved up to buy one.

Possible solutions for making wearables more affordable include exploring form factors of wearables that can be redesigned to be more affordable. Many wearables are overdesigned and engineered for sleekness, small size, and fashion, as well as feature sets that may not align with the needs of low-SES communities. In essence, the question is what specific features might be more useful than having a general-purpose platform that does it all (but therefore is much more expensive). Similar to how low-feature phones (text, phone call, and light browsing ability) are a 10th of the price of a smartphone, wearable devices must also explore this avenue of sacrificing features (like sophisticated GPUs and video streaming accelerators or advanced machine learning processing) for things like optical respiratory rate measurement.

From an academic perspective, as stated previously, including low-SES communities in research studies is important to diversify participant populations to reduce potential racial bias. However, wearable researchers can take a step further and allow participants from low-SES communities to keep any technology used within the research. Giving people technology for a 2-month deployment and then taking it away when they do not have the means to obtain something like that themselves is a serious ethical concern.

### Limitations and Future Directions

We acknowledge that our work has sampling limitations. This study was exploratory, and though our intentions were not meant to only target participants from Hispanic or Latine low-SES communities from 2 metropolitan cities, we were not able to sample the perceptions of other racially minoritized BIPOC communities from smaller cities or rural areas. City representation was likely due to the research team members being from these 2 cities. In future work, examining the differences in how members from other racially minoritized groups perceiving wearables could vary would provide valuable insights.

### Conclusions

This research study aimed to investigate the needs and perspectives of individuals from low-income communities regarding the adoption and usage of wearable devices. Our findings indicate that there is considerable interest among members of these communities in employing wearables to promote their well-being. Participants expressed frustration with the current health system citing how the lack of health resources and the health effects of the COVID-19 pandemic has led them to seek alternative methods to manage their health. Participants recommended design considerations for the utility of wearable devices that included, durability, sustainability, and accessibility. Additionally, autonomy on how their data is used was important for the majority of participants. Affordability was the primary barrier to the adoptability of wearable devices. Participants believe that if health insurance companies can help pay for partial costs of wearable devices, more people in the community would be more interested in using them. The insights from this study serve as a first step for researchers and technology companies in the domain of wearable technology to develop tools that account for the contextual and cultural perspectives of low-SES communities to help democratize the utility of wearable devices.

## Appendix

Multimedia Appendix 1 Standards for reporting qualitative research checklist.

Multimedia Appendix 2 Study consent form.

Multimedia Appendix 3 Interview guidelines and questions round 1.

Multimedia Appendix 4 Interview guidelines and questions round 2.

## Abbreviations

BIPOC: Black, Indigenous, people of color
LGBTQ+: lesbian, gay, bisexual, transgender, queer
mHealth: mobile health
SES: socioeconomic status
