# Supplementary material for: Perceptions of Wearable Health Tools Post the COVID-19 Emergency in Low-Income Latin Communities: Qualitative Study
Source: JMIR Mhealth Uhealth. 2024 May 8;12:e50826. doi: 10.2196/50826 (PMC11112471; doi:10.2196/50826)
Supplement: Multimedia Appendix 2 [file mhealth_v12i1e50826_app2.pdf]

Consent to Participate in Research

Title of Research Study: Equityware

Investigator:

Dr. Josiah Hester
Assistant Professor
Department of Computer Science
Department of Preventive Medicine
Northwestern University
2145 Sheridan Rd, Evanston IL, 60208
(847) 467-7571

Supported By: This research is supported by Northwestern University, Department of Electrical and Computer Engineering.

Key Information about this research study:

The following is a short summary of this study to help you decide whether you want to be a part of this study. Information that is more detailed is listed later in this form.

The purpose of this study is to understand underserved communities' perceptions and awareness of wearable computing devices and allow them to participate in the co-design of wearable devices that can be used to support them in their everyday lives.

You will be asked a series of questions in the form of a semi-structured interview over video call. Each interview will last one hour. We expect that you will take part in our study for a total of 1 hour. You will face no foreseeable risks. We expect around 15 to 20 people to participate in this research study. The main benefit of participating in this study is to expand our knowledge on if and how wearable technologies can be used to better support the wellbeing of underserved populations.

Why am I being asked to take part in this research study?

We are asking you to take part in this research study because you are an adult (above 18) who can consent and are considered to be from an underserved community.

How many people will be in this study?

We expect about 15 to 20 people will be in this research study.

What should I know about participating in a research study?

- Someone will explain the research study to you.
- Whether or not you take part is up to you.
- You can choose not to take part.
- You can agree to take part and later change your mind.
- Your decision will not be held against you.
- You can ask all the questions you want before you decide.

What happens if I say, "Yes, I want to be in this research"?

If you choose to participate in this study, you will be participating in a one-hour semi structured interview. The interviews are 1-1 with a graduate student from Northwestern University. Interviews will be conducted through Zoom. The research will be conducted from mid-December to early January. Interviews will be recorded. During the interview, you will be asked open-ended questions on your awareness, experience, and perception of wearable devices. We will ask questions if you have used any wearable devices in the past. If you have worn wearable devices or have knowledge of them, we will ask questions on how you have or would use the devices and what other applications you would use them for. We will ask for specifics of devices and apps, as well as examples of your usage in your everyday life.

Will being in this study help me in any way?

We cannot promise any benefits to you or others from your taking part in this research. However, possible benefits include increased awareness (e.g., challenges, opportunities) on how low-income minorities can benefit from using wearable devices from health, or other socio-economic perspectives.

Is there any way being in this study could be bad for me?

It is unlikely that the questions we ask will lead to any legal, social, or psychological problems. A possible risk for any research is that confidentiality could be compromised – that is, people outside the study might get hold of confidential study information. We will do everything we can to minimize this risk, as described in more detail later in this form.

What happens if I do not want to be in this research?

Participation in research is voluntary. You can decide to participate or not to participate.

What happens if I say "Yes", but I change my mind later?

You can leave the research at any time, and it will not be held against you.

What happens to the information collected for the research?

Efforts will be made to limit the use and disclosure of your personal information, including research study records, to people who have a need to review this information. We cannot promise complete secrecy. Organizations that may inspect and copy your information include the IRB and other representatives of this institution. The results of this study may also be used for teaching, publications, or for presentation at scientific meetings. If identifiers are removed from your identifiable private information or identifiable samples that are collected during this research, that information or those samples could be used for future research studies or distributed to another investigator for future research studies without your additional informed consent. If we learn about current or ongoing child or elder abuse or neglect, we may be required or permitted by law or policy to report this information to authorities.

Data Sharing: De-identified data from this study may be shared with the research community at large

to advance science and health. We will remove or code any personal information that could identify you before files are shared with other researchers to ensure that, by current scientific standards and

known methods, no one will be able to identify you from the information we share. Despite these measures, we cannot guarantee anonymity of your personal data.

What else do I need to know?

Compensation: If you agree to take part in this research study, we will pay you $40 for your time and effort. If you withdraw, you will not receive compensation.

You will be paid with a $40 Gift Card at the end of the study.

Who can I talk to?

If you have questions, concerns, complaints, or think the research has affected you in some way, talk to the research team at (847) 467-7571.

This research has been reviewed and approved by an Institutional Review Board ("IRB"). You may talk to them at (312) 503-9338 or irb@northwestern.edu if:

- Your questions, concerns, or complaints are not being answered by the research team.
- You cannot reach the research team.
- You want to talk to someone besides the research team.
- You have questions about your rights as a research participant.
- You want to get information or provide input about this research.

Signature for Adult 18 or older

Your signature documents your permission to take part in this research.

____ ____________________

Signature of participant Date

____

Printed name of participant

____ ____________________

Signature of person obtaining consent Date

____

Printed name of person obtaining consent

Document

Template Revision Date: 4-6-2018
