# Supplementary material for: Perceptions of Wearable Health Tools Post the COVID-19 Emergency in Low-Income Latin Communities: Qualitative Study
Source: JMIR Mhealth Uhealth. 2024 May 8;12:e50826. doi: 10.2196/50826 (PMC11112471; doi:10.2196/50826)
Supplement: Multimedia Appendix 3 [file mhealth_v12i1e50826_app3.docx]

**Multimedia Appendix 2.** Preliminary investigation questions: understanding community needs.

(1) Do you own a mobile phone?

(2) Do you rely on other facilities to have access to Wi-Fi (ex.cofee shop, school, library? If Wi-Fi is not available, what are other wireless connectivities do you have access to (e.g., cellular data?)

(3) Are you able to charge your phone in your home or do you rely on other facilities to charge your phone?

(4) What are your resource constraints if you have any?

(5) What are your thoughts on wearable technology? Are people willing to adopt this technology or is it frowned upon?

(6) Do you own a wearable device? If so, what is it? How often do you use it? What applications do you use it for?

(7) Would you be comfortable using wearable technology?

(8) What would make a wearable device feel uncomfortable for you? How would the size and/or weight of a wearable device impact your decision on whether you find it comfortable or not? What about the cost of a wearable device? Would you find expensive wearable devices too intimidating to use? What about appearance?

(9) If you could design a wearable device that would ft your needs, what functionalities would you want it to have? How would you want it to look like?

(10) Do you think people in your community would benefit from this wearable device?
