# Supplementary material for: Perceptions of Wearable Health Tools Post the COVID-19 Emergency in Low-Income Latin Communities: Qualitative Study
Source: JMIR Mhealth Uhealth. 2024 May 8;12:e50826. doi: 10.2196/50826 (PMC11112471; doi:10.2196/50826)
Supplement: Multimedia Appendix 4 [file mhealth_v12i1e50826_app4.docx]

**Secondary Investigation Interview Guidelines and Questions:**

## Introduction + Consent [5 min]

<Read Aloud:

Thanks so much for taking the time to participate! My name is [your name] and I am a researcher in the [lab name at Northwestern University].

Before we begin, some important things we want to reiterate:

- You will be compensated with a $40 Gift Card via Northwestern for participating.
- We’ll start with a brief interview and spend most of the time on feedback for some ideas.
- All data collected will be anonymized, used for research purposes only, and held in a secure location only accessible through 2factor authentication with our university credentials.
- During our conversation, try not to use specific names, organizations, or places if you can; instead you could refer to them as “Person A, Organization B, large city, etc.”
- You can choose not to answer any question or prompt if you’re uncomfortable or for whatever reason don’t wish to share, that’s totally okay. We can also go a little slower or you can ask to completely stop the session.
- <If they have not changed their name: Since we’d like to record this session, would you like to change your screen name? <Give participants the ability to change their username before proceeding. Directions: Go to “Participants” at the bottom > Participant Panel on the right should appear > Click on their own name > New window should appear where they can type in their name > Click “Yes” to confirm change>
- Once again, we’d like to confirm. Do we have your consent to record?
- <If yes: Please state that you give your consent for us to conduct, record, and take notes of this session after you hear “This meeting will be recorded”.> /end Read Aloud>

Interview Questions

#### ***Questions About The Neighborhood***

1. Can you tell me a bit about the neighborhood or area you live in? Are there things you like and dislike about it?
   1. How do you feel about the general health and wellbeing of people in your neighborhood community?
      1. <If not mentioned: How has COVID impacted you and your community? Have there been any lasting impacts?>

Great, thanks for telling me about your neighborhood! I’d now love to talk about the safety in your neighborhood:

1. What activities do you feel safe doing in your neighborhood? (Like going for a walk, hanging out at a park, etc.)
   1. Are there activities you don’t feel safe doing in your neighborhood area?
2. How do you feel about walking alone around your neighborhood?
   1. What about when you’re walking alone at night versus during the day?
   2. What concerns do you have, if any, about what could happen to you or someone you know when walking alone?
3. Have you ever felt unsafe in your area or in unfamiliar places (like other neighborhoods, stores, bars, or when doing things like going shopping, visiting other people, etc.)?
   1. What do you do when you’re feeling unsafe?
   2. Do you carry anything with you to help you feel more safe?
4. Have you or someone you know ever been harassed or attacked in your neighborhood or unfamiliar area? If you’re comfortable sharing, could you tell me a bit more about what happened?
   1. Were authorities like police or some other 3rd party notified? *<If not shared: Did you feel like they were helpful in that situation?>*
   2. How did you feel about that experience or hearing about what happened?

*<Reminder to validate participants’ experiences & thank them for sharing their time to help us try to address this issue>*

1. What advice do you or would you give to a loved one who was going to be walking alone in an unfamiliar or unsafe area?

Thank you for all that helpful information! I now want to talk about technology for a bit and I’m specifically curious about your thoughts of wearable devices. So by that I mean something that you wear on your body as an accessory that collects information like your health or movement.

#### **Questions About Health & Wearables**

So to start:

1. What are your thoughts on wearable devices in general?
2. Are there any specific wearable devices that you have or know about?
   1. <for each one they list ask the following questions>
      1. What do you like about it?
      2. What do you dislike about it?
      3. What do you use it for?
      4. Are there any other wearable devices that you use?
3. What questions or concerns do you have about your health and COVID-19, or just your health more broadly?
   1. What information would be useful for you to know about your own health?
   2. Could you think of a way that wearable devices could be helpful with COVID?

Okay great, thank you. These were great ideas! Now I’m going to share a couple scenarios of ideas that we had about how we could use wearable devices to help us feel safer. And I would love your feedback on these ideas!
